# Distinct Time-Course of Alterations of Groups I and II Metabotropic Glutamate Receptor and GABAergic Receptor Expression Along the Dorsoventral Hippocampal Axis in an Animal Model of Psychosis

**DOI:** 10.3389/fnbeh.2019.00098

**Published:** 2019-05-08

**Authors:** Valentyna Dubovyk, Denise Manahan-Vaughan

**Affiliations:** ^1^Department of Neurophysiology, Medical Faculty, Ruhr-University Bochum, Bochum, Germany; ^2^International Graduate School of Neuroscience, Ruhr-University Bochum, Bochum, Germany

**Keywords:** psychosis, MK801, hippocampus, dorsoventral axis, mGlu receptors, GABA receptors, rodent

## Abstract

Psychosis is a clinical state that encompasses a range of abnormal conditions, including distortions in sensory information processing and the resultant delusional thinking, emotional discordance and cognitive impairments. Upon developing this condition, the rate at which cognitive and behavioral deteriorations progress steadily increases suggesting an active contribution of the first psychotic event to the progression of structural and functional abnormalities and disease establishment in diagnosed patients. Changes in GABAergic and glutamatergic function, or expression, in the hippocampus have been proposed as a key factor in the pathophysiology of psychosis. However, little is known as to the time-point of onset of putative changes, to what extent they are progressive, and their relation to disease stabilization. Here, we characterized the expression and distribution patterns of groups I and II metabotropic glutamate (mGlu) receptors and GABA receptors 1 week and 3 months after systemic treatment with an *N*-methyl-D-aspartate receptor (NMDAR) antagonist (MK801) that is used to model a psychosis-like state in adult rats. We found an early alteration in the expression of mGlu1, mGlu2/3, and GABA_B_ receptors across the hippocampal dorsoventral and transverse axes. This expanded to include an up-regulation of mGlu5 levels across the entire CA1 region and a reduction in GABA_B_ expression, as well as GAD67-positive interneurons particularly in the dorsal hippocampus that appeared 3 months after treatment. Our findings indicate that a reduction of excitability may occur in the hippocampus soon after first-episode psychosis. This changes, over time, into increased excitability. These hippocampus-specific alterations are likely to contribute to the pathophysiology and stabilization of psychosis.

## Introduction

Schizophrenia is a chronic mental disorder, distinguished by a rich spectrum of symptoms that are clustered into the cognitive, negative, and psychotic domains (McGhie and Chapman, 1961; Kapur, 2003; Bowie and Harvey, 2005; Bürgy, 2008; Velligan and Alphs, 2008; Davis et al., 2014). Whilst a decline in learning and memory ability (cognitive symptoms) and social withdrawal (negative symptoms) become apparent early on, in the course of the disease (Keshavan et al., 2011; Keefe and Harvey, 2012), it is the manifestation of hallucinatory and delusionary experiences (psychotic symptoms) that lead to clinical diagnosis. Following the first major psychotic event, also known as the first-episode of psychosis (Larson et al., 2010; Keshavan et al., 2011; Ruby et al., 2014), the rate at which cognitive and behavioral deteriorations occur progressively increases (Häfner et al., 1994; Cheung et al., 2010; Alvarez-Jimenez et al., 2011). This suggests an active involvement of the first-episode of psychosis in the progression of structural and functional abnormalities in diagnosed patients (Borgwardt et al., 2008; Haijima et al., 2012; Guo et al., 2013).

It has been proposed that a dysfunction of the glutamatergic and GABAergic systems are key elements to the etiology of schizophrenia and psychosis (Belforte et al., 2010; Nakazawa et al., 2012; Snyder and Gao, 2013). In particular, it is believed that hypofunction of *N*-methyl-D-aspartate receptors (NMDAR) on GABAergic interneurons, particularly on parvalbumin-containing (PV) interneurons, leads to disinhibition of glutamatergic terminals and a hyperglutamatergic state (Marek et al., 2010; Riebe et al., 2016). This disinhibition further results in functional deficits of neurons, circuits and behavior that are characteristic of psychosis and can be emulated by NMDAR antagonists (Krystal et al., 1994; Lahti et al., 1995), and in transgenic animal models of NMDAR hypofunction (Belforte et al., 2010).

Importantly, reduced inhibitory and increased excitatory neurotransmission may, in turn, modulate the function of other glutamatergic and GABAergic receptors, as well as be modulated by them. Here, the (group I) metabotropic glutamate receptor 5 (mGlu5) is of prime interest as it shares a unique interrelationship, both in terms of physical coupling and functional modulation, with NMDAR (Tu et al., 1999; Marino and Conn, 2002; Alagarsamy et al., 2005). Another group I mGlu receptor, mGlu1, potentiates NMDAR currents (Benquet et al., 2002; Heidinger et al., 2002). Furthermore, mGlu1 and mGlu5 receptors both facilitate long-term potentiation (LTP) in the hippocampus (Mukherjee and Manahan-Vaughan, 2013), a brain region that is among the most affected in patients suffering from schizophrenia and psychosis (Heckers and Konradi, 2002; Kolomeets et al., 2007; Adriano et al., 2012; Yin et al., 2012). Group II mGlu receptors, mGlu2 and mGlu3, serve as autoreceptors at glutamatergic terminals (Shigemoto et al., 1997; Schoepp, 2001) and are critically required for persistent forms of hippocampal long-term depression (LTD) (Kobayashi et al., 1996; Manahan-Vaughan, 1998; Pöschel and Manahan-Vaughan, 2007; Altinbilek and Manahan-Vaughan, 2009). Thus, both group I and group II receptors play an intrinsic role in the regulation and support of NMDAR-dependent information processing and storage, as well as in the regulation of activity-dependent neural excitability.

GABA is the primary inhibitory neurotransmitter in the mammalian brain. The ionotropic GABA_A_ receptor is a ligand-gated chloride channel that is responsible for tonic inhibition in the brain. Because of their high affinity for GABA, along with their relatively slow desensitization rates, it has been proposed that GABA_A_ receptors sense both the ambient concentration of GABA, as well as the activity- dependent spill-over of GABA from the synaptic cleft (Wlodarczyk et al., 2013). The GABA_B_ receptor is a metabotropic receptor that serves as an autoreceptor (Waldmeier et al., 2008), but also can modulate GABAergic responses of neurons (Shen et al., 2017). The efficacy of both receptors is regulated by synaptic activity: activation of calcium calmodulin kinase II (CAMKII), a downstream target of NMDAR activation, results in phosphorylation of GABA_B_ receptors (Zemoura et al., 2018). Furthermore, both protein kinase A (PKA) and protein kinase C (PKC) can mediate phosphorylation of GABA_A_ receptors (Connelly et al., 2013). GABA_A_ and GABA_B_ receptors serve to keep local cellular and network excitation within a functional physiological range and enable the precise spatiotemporal conditions that are required for information encoding through synaptic plasticity (Kaila, 1994; Paulsen and Moser, 1998; Bettler et al., 2004; Kullmann et al., 2005). A disruption of GABAergic inhibitory control can, thus, be expected not only to have a potent effect at the level of tonic inhibition, but also at the level of excitation-inhibition balance. The effectivity of information processing related to cognition, learning and memory will consequently be compromised.

The hippocampus is a critical processing hub for a wide range of sensory and cognitive information (Kesner and Rolls, 2015) and is a primary site for activity-dependent synaptic plasticity that in turn enables both short- and long-term declarative memory (Kemp and Manahan-Vaughan, 2007). Patients with schizophrenia and psychosis exhibit a wide range of sensory and cognitive abnormalities (McGhie and Chapman, 1961; Kapur, 2003; Bowie and Harvey, 2005; Bürgy, 2008) that have been ascribed, at least in part, to dysfunctions in sensory information processing by the hippocampus (Arnold, 1999; Bast and Feldon, 2003; Behrendt, 2010; Bak et al., 2014; Kalweit et al., 2017). Not only is the trisynaptic circuit [dentate gyrus (DG) and CA regions (CA3 and CA1)] of the hippocampus responsible for the formation of multimodal memory representations (Hasselmo, 2005; Kesner, 2013a,b; Kesner and Rolls, 2015), but its longitudinal (dorso-ventral) axis is pivotal to the processing of visuo-spatial and socio-emotional elements of cognition (Bast et al., 2009; Fanselow and Dong, 2010; Strange et al., 2014). Strikingly, changes in GABAergic and groups I and II mGlu receptor function or expression in the hippocampus have been proposed to form a key factor in psychotic events in schizophrenia (Linden et al., 2006; Behrendt, 2010; Matosin et al., 2015; Zurawek et al., 2018). Nonetheless, little is known as to the time-point of onset of putative changes on these receptor systems, or to what extent changes are progressive during the course of chronification of the disease. Evidence for early changes in receptor function, soon after the first-episode of psychosis could offer novel possibilities for therapy and disease intervention.

In the present study our goal was to study putative changes in the abovementioned receptor systems by examining receptor expression in a rodent model of first-episode psychosis. Here, an acute systemic injection of MK801, an NMDAR antagonist, was used to mimic the first-episode of psychosis (Manahan-Vaughan et al., 2008a; Wiescholleck and Manahan-Vaughan, 2013a,b). Rodent features of a psychosis-like episode comprise significant deficits in prepulse inhibition of the acoustic startle response and startle inhibition, increased stereotypy and hyperactivity (Manahan-Vaughan et al., 2008a; Wiescholleck and Manahan-Vaughan, 2013a) that parallel symptoms exhibited by patients during the first episode of psychosis (Ludewig et al., 2003; Kim et al., 2011; Compton et al., 2015).

The first episode-like event in rodents is followed by a persistent loss of hippocampal LTP, deficits in hippocampus-dependent learning and memory (Manahan-Vaughan et al., 2008b; Wiescholleck and Manahan-Vaughan, 2013b; Wlodarczyk et al., 2013), changes in hippocampal neuronal oscillations (Kehrer et al., 2007; Kalweit et al., 2017), persistent elevations of neuronal excitability and somatic immediate early gene expression (Grüter et al., 2015). In patients, overt changes in brain function comprise decreases in hippocampal functional connectivity and deficits in relational memory (Samudra et al., 2015; Avery et al., 2018), increased cortical excitability (Díez et al., 2017), and abnormal neural oscillations (Uhlhaas and Singer, 2010). These changes form the fundament of the “disconnection hypothesis” (Friston, 1998; Díez et al., 2017) and are widely believed to be caused by a combination of focal brain changes and a disruption of synaptic plasticity that leads to impairments of functional integration at the cognitive level (Stephan et al., 2006, 2009). As mentioned above, changes in GABA and glutamate receptor function have been proposed to underlie these changes (Stephan et al., 2006, 2009; Corlett et al., 2011; Gonzalez-Burgos and Lewis, 2012). In line with this, changes in expression of NMDAR subunits along the dorsoventral axis of the hippocampus have been reported in a rodent model of psychosis (Dubovyk and Manahan-Vaughan, 2018a). Less is known about changes in GABA receptors, or subunits of mGlu receptors that may accompany disease progression.

We characterized the expression and distribution patterns of metabotropic glutamatergic and GABAergic receptors 1 week and 3 months after the emulation of the first psychotic event in adult rats. In particular, we looked for changes in the expression of mGlu1, mGlu2/3, mGlu5, GABA_A_, and GABA_B_ receptors across the laminar structure of the trisynaptic circuit and along the longitudinal axis of the hippocampus. We detected time-dependent and neural subcompartment-specific changes in the expression of these receptors across the longitudinal axis of the hippocampus. Our findings indicate that an early adaptive reorganization of the hippocampus occurs after first-episode psychosis that contributes to a reduction of network excitation, and is superseded by a state of permanently increased network excitability and reduced interneuron number. These changes are likely to support loss of synaptic gain control that have been proposed by others (Adams et al., 2013; Díez et al., 2017) to contribute importantly to the pathophysiology and stabilization of psychosis.

## Materials and Methods

### Animals

All experiments were done with healthy male Wistar rats (Charles River Laboratories, Germany). Animals were housed in custom-made climatised and ventilated holding cupboards in an animal-housing room with a controlled 12-h light/dark cycle. No female rats were housed in the room. Animals had free access to food and water. The study was carried out in accordance with the European Communities Council Directive of September 22nd, 2010 (2010/63/EU) for care of laboratory animals. Prior permission was obtained from the ethics commission of the local government authority (Landesamt für Arbeitsschutz, Umwelt- und Naturschutz, Nordrhein-Westfalen).

### Drug Treatment

Seven-to-eight-week-old Wistar rats were divided into two groups where each animal received a single intraperitoneal (i.p.) injection of the NMDAR antagonist [+]-5-methyl-10,11-dihydro-5Hdibenzo-[a,d]-cyclohepten-5,10-imine hydrogen maleate (MK801, Tocris, Germany). The compound was dissolved in 0.9% physiological saline and administered in a concentration of 5 mg/kg, in accordance with previous studies conducted by our group (Manahan-Vaughan et al., 2008a,b). This treatment protocol results in a first-episode-like state in rodents (Wöhrl et al., 2007; Manahan-Vaughan et al., 2008a,b, 2013a,b; Wiescholleck and Manahan-Vaughan, 2012). Two control animal groups of identical age and strain received a single i.p. injection of 0.9% physiological saline (10 ml/kg). Animals were sacrificed 1 week or 3 months after the injection. Each group included 5 animals, with the exception of the 3-month-group where 3 additional animals were added to confirm a strong statistical tendency in the original dataset. From each animal, one section of the DH, IH, or VH was analyzed with regard to a specific receptor. Both, right and left hippocampi were used for the analysis and considered as replicates.

### Slice Preparation

**FIGURE 1:**
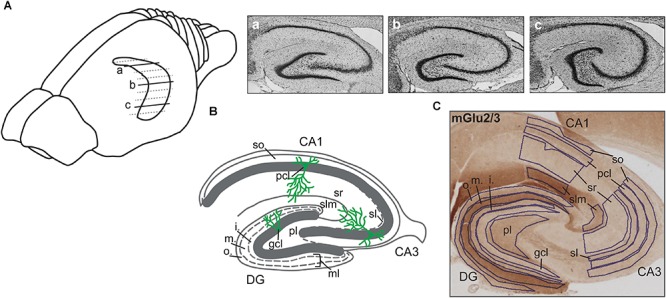
Illustration of hippocampal sections. **(A)** Drawing of the rat brain showing the horizontally sectioned hippocampus along its longitudinal axis. Nissl-stained sections correspond to dorsal **(a)**, intermediate **(b)**, and ventral **(c)** subdivisions. **(B)** Schematic illustration of the hippocampal transverse axis depicting its trisynaptic circuit with the laminar view. o., outer band of the ml of the DG; m., middle band of the ml of the DG; i., inner band of the ml of the DG; gcl, granule cell layer of the DG; ml, molecular layer of the DG; pl, polymorphic layer of the DG; so, Stratum oriens of CA3/CA1; pcl, pyramidal cell layer of CA3/CA1; sl, Stratum lucidum of the CA3; sr, Stratum radiatum of CA3/CA1; and slm, Stratum lacunosum-moleculare of CA3/CA1. **(C)** A representative example of the dorsal hippocampus showing the expression of mGlu2/3 receptors across its transverse axis. Laminal delineation across the DG, CA3, and CA1 regions is shown.

Wistar rats were deeply anesthetized with sodium pentobarbital and transcardially perfused with cold Ringer’s solution + heparin (0.2%) followed by 4% paraformaldehyde (PFA) in phosphate buffered saline (PBS, 0.025 M). Brains were removed, fixed in 4% PFA for 24 h, and cryoprotected in 30% sucrose in 0.1 M PBS for at least 3 days. Serial 30-μm thick horizontal sections were collected with a freezing microtome. For each animal, three horizontal sections from the most dorsal (between 3.6 and 4.1 mm posterior to bregma), middle intermediate (around 5.6 mm posterior to bregma) and most ventral hippocampal parts (between 7.1 and 7.6 mm posterior to bregma) were simultaneously used for immunohistochemical staining (Figure 1A). Sections from the Vehicle- and MK801-treated animals were always analyzed in pairs.

### Immunohistochemistry

Antibody specificity was verified in our previous studies (Grüter et al., 2015; Dubovyk and Manahan-Vaughan, 2018a,b). Endogenous peroxidase was blocked by pretreating the free-floating brain sections in 0.3% H_2_O_2_ for 20 min. They were then rinsed in PBS and incubated with blocking solution containing 10% normal serum and 20% avidin in PBS with 0.2% Triton X-100 (PBS-Tx) for 90 min at room temperature (RT). Sections were incubated overnight at room temperature with primary antibodies: mGlu2/3 (rabbit polyclonal, 1:200; ab1553, Chemicon), mGlu5 (rabbit polyclonal, 1:200; ab5675, Millipore), GABA_A_ receptor (mouse monoclonal, 1:400; mab341, Millipore), GABA_B_ receptor 1 (mouse monoclonal, 1:250; ab55051, Abcam) or GAD67 (mouse monoclonal, 1:100; mab-5406, Millipore) in medium containing 1% normal serum in 0.2% PBS-Tx + 20% biotin. Sections were then rinsed in PBS and incubated with biotinylated goat anti-rabbit (1:500; BA-1000, Vector), horse anti-mouse (1:500; BA-2001, Vector) or horse anti-goat (1:500; BA-9500, Vector) antibodies in 1% normal serum in 0.1% PBS-Tx for 90 min at RT. Afterwards, sections were washed in PBS and incubated for 90 min at RT with ABC kit (PK-6100, Vector) in 1% normal serum in 0.1% PBS-Tx.

Staining with mGlu1 receptor antibodies required an additional amplification step with biotinylated tyramide for 20 min. Here, the sections were incubated for 5 days at 4°C with primary mGlu1 receptor antibodies (rabbit polyclonal, 1:400; ab82211, Abcam) in 1% BSA in 0.2% TBS-Tx. PBS was replaced with TBS, normal serum + PBS-Tx with bovine serum albumin + TBS-Tx, and one ABC reaction with two for 30 min each with amplification step in between. Here, sections were incubated with 10 μl b-tyramide + 10 μl 0.01% H_2_O_2_ in 1,000 μl of TBS for 20 min. Finally, the sections were washed in PBS and treated with diaminobenzidine and 0.01% H_2_O_2_ for approximately 10 min.

### Quantitative Analysis

Regions of interest were defined using the rat brain atlas of Paxinos and Watson (1982) and Nissl staining where every 12th section throughout the whole hippocampus was stained with 0.1% Cresyl violet (c5042, Sigma) as a reference. Eleven areas of interest included: molecular layer (ml) of the dentate gyrus (DG); granule cell layer (gcl) of the DG; polymorphic layer (pl) of the DG; Stratum oriens (so) of CA3/CA1; pyramidal cell layer (pcl) of CA3/CA1; Stratum radiatum (sr) of CA3/CA1; and Stratum lacunosum-moleculare (slm) of CA3/CA1 on sections taken from the dorsal, intermediate and ventral hippocampal subdivisions. Due to the expression profile of mGlu2/3 receptors, a specific delineation of the ml of the DG into outer, middle and inner bands was possible. The Stratum lucidum (sl) of the CA3 could also be clearly identified and was therefore quantified, resulting in 15 areas of interest for the mGlu2/3 receptor (Figure 1B,C). Importantly, for background subtraction we used receptor-devoid tissue, which in the dorsal sections was the fimbria; in the intermediate sections was either the fimbria or the superior thalamic radiation; and in the ventral sections was the internal capsule. Pictures of stained sections were acquired with a light microscope (Leica DMR, Germany), equipped with a digital camera (MBF Bioscience) and stored in TIFF format. The regions of interest were analyzed at 2.5× lens magnification. The digitized high-resolution pictures were obtained using Neurolucida software (MBF Bioscience) and quantified using open-source ImageJ software (National Institutes of Health). Given that images were acquired with a RGB camera the ‘Color Deconvolution’ plugin in ImageJ was used to deconvolve the color information as well as to convert images to 8-bit format, thus increasing the dynamic range of the signal. R software was used to scale data from several independent stainings/plates using generalized residual sum of squares algorithm to account for batch variability in staining intensities (Kreutz et al., 2007; von der Heyde et al., 2014).

The amount of GAD67 positive cells was manually calculated in sections stained with anti-GAD67 antibodies. Quantification was limited to the DG, CA3, and CA1 regions across their respective laminal structures of the DH, IH, and VH sections.

### Statistical Analysis

Data obtained in the immunohistochemical experiments were statistically analyzed by means of factorial analysis of variance (ANOVA) followed by Duncan’s *post hoc* test, which allowed detection of significant factors in a two-factor model. Here, the Vehicle and MK801 treatment data were considered as the TREATMENT factor (factor one), while the laminar organization of the trisynaptic circuit as the REGION factor (factor two). The cell count results were analyzed using the Students *t*-test. All significant differences were defined as *p* < 0.05 or *p* < 0.01. Values are expressed as mean values ± the standard error of the mean (SEM).

## Results

To allow for detailed scrutiny of the hippocampus we studied the following hippocampal subcompartments: the molecular layer (ml), the granule cell layer (gcl), and the polymorphic layer (pl) of the dentate gyrus (DG), as well as the Stratum oriens (so), pyramidal cell layer (pcl), Stratum radiatum (sr), and Stratum lacunosum-moleculare (slm) of CA3/CA1. Sections from the dorsal hippocampus (DH), intermediate hippocampus (IH), and ventral hippocampus (VH) were examined (Figure 1). Expression of mGlu1, mGlu5, mGlu2/3, GABA_A_, and GABA_B_ receptors was examined 1 week and 3 months after treatment with vehicle, or the NMDAR antagonist, MK801.

### MGlu1 Expression Is Altered 1 Week, but Not 3 Months After MK801 Administration

**FIGURE 2:**
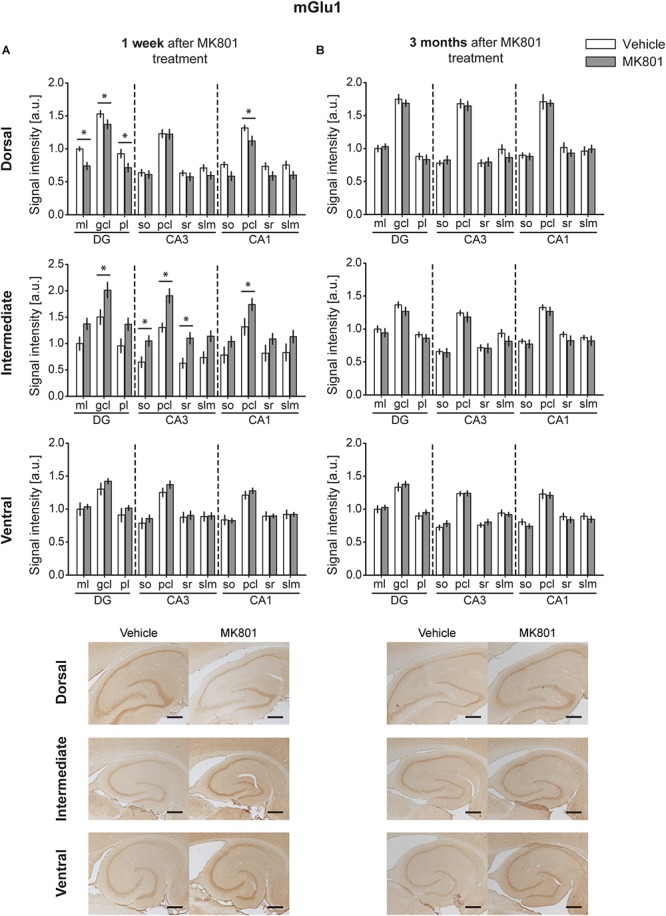
MK801 treatment leads to a transient change of mGlu1 receptor protein expression. Bar charts represent relative changes in protein levels 1 week and 3 months after MK801 treatment across the somato-dendritic layers of the transverse and longitudinal hippocampal axes. **(A)** mGlu1 receptor levels were (*down-regulated in the dorsal hippocampus, but up-regulated in the intermediate hippocampus 1 week after MK801 treatment. **(B)** No changes in mGlu1 receptor expression were detected 3 months after MK801 administration. Scale bar: 500 μm. Values expressed in arbitrary units (a.u.). Error bars indicate SEM. ^∗^*p* < 0.05 or ^∗∗^*p* < 0.01. ml, molecular layer of the DG; gcl, granule cell layer of the DG; pl, polymorphic layer of the DG; so, Stratum oriens of CA3/CA1; pcl, pyramidal cell layer of CA3/CA1; sr, Stratum radiatum of CA3/CA1; and slm, Stratum lacunosum-moleculare of CA3/CA1. Photomicrographs provide examples of mGlu1 receptor-stained sections from the dorsal, intermediate and ventral hippocampal subdivisions that originated from the same vehicle- or MK801-treated animal and correspond to 1 week (left) or 3 months (right) after treatment.*)

One week after MK801 treatment, mGlu1 receptor protein levels were significantly changed in DH, and IH [multifactorial ANOVA, Treatment factor, DH: *F*(_1,198_) = 32.364, *p* < 0.001; IH: *F*(_1,161_) = 58.727, *p* < 0.001]. In the DH, protein expression was reduced in the ml, gcl, and pl of the DG and in the pcl of the CA1 (Figure 2A and Table 1). In contrast, the IH showed an up-regulation of mGlu1 receptor levels in the gcl of the DG, so, pcl and sr of the CA3; and pcl of the CA1. The VH remained unaltered [multifactorial ANOVA, Treatment factor, VH: *F*(_1,165_) = 3.209, *p* = 0.075] (Figure 2A and Table 1).

**Table 1:** An overview of statistically significant differences in the expression of metabotropic glutamatergic receptors.

Receptor	Time-point	Hippocampal axis part	Multifactorial ANOVA			
			TREATMENT factor (T)	REGION factor (R)	T ^∗^ R interaction	Duncan’s *post hoc* test
mGlu1	1 week	DH	*F*(_1,198_) = 32.364,	*F*(_10,198_) = 55.487,	*F*(_10,198_) = 0.968,	**ml/DG: *p* = 0.002**
			***p* < 0.001**	***p* < 0.001**	*p* = 0.472	**gcl/DG: *p* = 0.047**
						**pl/DG: *p* = 0.017**
						so/CA3: *p* = 0.748
						pcl/CA3: *p* = 0.909
						sr/CA3: *p* = 0.557
						slm/CA3: *p* = 0.218
						so/CA1: *p* = 0.069
						**pcl/CA1:** ***p* = 0.023**
						sr/CA1: *p* = 0.127
						slm/CA1: *p* = 0.111
		IH	*F*(_1,161_) = 58.727,	*F*(_10,161_) = 14.245,	*F*(_10,161_) = 0.354,	ml/DG: *p* = 0.077
			***p* < 0.001**	***p* < 0.001**	*p* = 0.963	**gcl/DG: *p* = 0.005**
						pl/DG: *p* = 0.052
						**so/CA3: *p* = 0.048**
						**pcl/CA3: *p* = 0.001**
						**sr/CA3: *p* = 0.021**
						slm/CA3: *p* = 0.053
						so/CA1: *p* = 0.208
						**pcl/CA1: *p* = 0.027**
						sr/CA1: *p* = 0.187
						slm/CA1: *p* = 0.146
		VH	*F*(_1,165_) = 3.209,	*F*(_10,165_) = 18.678,	*F*(_10,165_) = 0.286,	ml/DG: *p* = 0.714
			*p* = 0.075	***p* < 0.001**	*p* = 0.983	gcl/DG: *p* = 0.224
						pl/DG: *p* = 0.309
						so/CA3: *p* = 0.49
						pcl/CA3: *p* = 0.246
						sr/CA3: *p* = 0.784
						slm/CA3: *p* = 0.931
						so/CA1: *p* = 0.908
						pcl/CA1: *p* = 0.502
						sr/CA1: *p* = 0.989
						slm/CA1: *p* = 0.996
	3 months	DH	*F*(_1,198_) = 0.885,	*F*(_10,198_) = 76.594,	*F*(_10,198_) = 0.398,	ml/DG: *p* = 0.737
			*p* = 0.347	***p* < 0.001**	*p* = 0.946	gcl/DG: *p* = 0.507
						pl/DG: *p* = 0.617
						so/CA3: *p* = 0.605
						pcl/CA3: *p* = 0.684
						sr/CA3: *p* = 0.844
						slm/CA3: *p* = 0.21
						so/CA1: *p* = 0.854
						pcl/CA1: *p* = 0.807
						sr/CA1: *p* = 0.407
						slm/CA1: *p* = 0.742
		IH	*F*(_1,187_) = 6.962,	*F*(_10,187_) = 36.306,	*F*(_10,187_) = 0.195,	ml/DG: *p* = 0.454
			***p* < 0.01**	***p* < 0.001**	*p* = 0.996	gcl/DG: *p* = 0.233
						pl/DG: *p* = 0.492
						so/CA3: *p* = 0.838
						pcl/CA3: *p* = 0.41
						sr/CA3: *p* = 0.924
						slm/CA3: *p* = 0.198
						so/CA1: *p* = 0.584
						pcl/CA1: *p* = 0.424
						sr/CA1: *p* = 0.282
						slm/CA1: *p* = 0.555
		VH	*F*(_1,194_) = 0.03,	*F*(_10,194_) = 45.12,	*F*(_10,194_) = 0.54,	ml/DG: *p* = 0.687
			*p* = 0.866	*p* < 0.001	*p* = 0.863	gcl/DG: *p* = 0.482
						pl/DG: *p* = 0.433
						so/CA3: *p* = 0.391
						pcl/CA3: *p* = 0.906
						sr/CA3: *p* = 0.472
						slm/CA3: *p* = 0.705
						so/CA1: *p* = 0.366
						pcl/CA1: *p* = 0.742
						sr/CA1: *p* = 0.451
						slm/CA1: *p* = 0.504
mGlu2/3	1 week	DH	*F*(_1,238_) = 5.687,	*F*(_13,238_) = 46.561,	*F*(_13,238_) = 0.969,	o./DG: *p* = 0.839
			***p* < 0.05**	***p* < 0.001**	*p* = 0.482	m./DG: *p* = 0.563
						i./DG: *p* = 0.936
						gcl/DG: *p* = 0.317
						pl/DG: *p* = 0.897
						so/CA3: *p* = 0.11
						pcl/CA3: *p* = 0.29
						sl/CA3: *p* = 0.22
						sr/CA3: *p* = 0.118
						slm/CA3: *p* = 0.23
						so/CA1: *p* = 0.483
						pcl/CA1: *p* = 0.583
						sr/CA1: *p* = 0.297
						slm/CA1: *p* = 0.097
		IH	*F*(_1,238_) = 18.317,	*F*(_13,238_) = 68.422,	*F*(_13,238_) = 1.28,	o./DG: *p* = 0.552
			***p* < 0.001**	***p* < 0.001**	*p* = 0.225	m./DG: *p* = 0.91
						i./DG: *p* = 0.604
						gcl/DG: *p* = 0.216
						pl/DG: *p* = 0.524
						**so/CA3: *p* = 0.047**
						**pcl/CA3: *p* = 0.044**
						sl/CA3: *p* = 0.118
						**sr/CA3: *p* = 0.016**
						slm/CA3: *p* = 0.064
						so/CA1: *p* = 0.374
						pcl/CA1: *p* = 0.315
						sr/CA1: *p* = 0.253
						slm/CA1: *p* = 0.076
		VH	*F*(_1,238_) = 36.241,	*F*(_13,238_) = 35.252,	*F*(_13,238_) = 0.4,	o./DG: *p* = 0.539
			***p* < 0.001**	***p* < 0.001**	*p* = 0.968	m./DG: *p* = 0.28
						i./DG: *p* = 0.215
						gcl/DG: *p* = 0.195
						pl/DG: *p* = 0.181
						**so/CA3: *p* = 0.03**
						pcl/CA3: *p* = 0.052
						**sl/CA3: *p* = 0.037**
						**sr/CA3: *p* = 0.039**
						slm/CA3: *p* = 0.062
						so/CA1: *p* = 0.275
						pcl/CA1: *p* = 0.233
						sr/CA1: *p* = 0.306
						slm/CA1: *p* = 0.567
	3 months	DH	*F*(_1,252_) = 9.8,	*F*(_13,252_) = 145.45,	*F*(_13,252_) = 0.27,	o./DG: *p* = 0.267
			***p* < 0.01**	***p* < 0.001**	*p* = 0.995	m./DG: *p* = 0.407
						i./DG: *p* = 0.252
						gcl/DG: *p* = 0.286
						pl/DG: *p* = 0.465
						so/CA3: *p* = 0.405
						pcl/CA3: *p* = 0.472
						sl/CA3: *p* = 0.483
						sr/CA3: *p* = 0.256
						slm/CA3: *p* = 0.668
						so/CA1: *p* = 0.8
						pcl/CA1: *p* = 0.862
						sr/CA1: *p* = 0.589
						**slm/CA1: *p* = 0.041**
		IH	*F*(_1,252_) = 0.00,	*F*(_13,252_) = 195.77,	*F*(_13,252_) = 0.19,	o./DG: *p* = 0.551
			*p* = 0.961	***p* < 0.001**	*p* = 0.999	m./DG: *p* = 0.539
						i./DG: *p* = 0.901
						gcl/DG: *p* = 0.551
						pl/DG: *p* = 0.717
						so/CA3: *p* = 0.75
						pcl/CA3: *p* = 0.741
						sl/CA3: *p* = 0.882
						sr/CA3: *p* = 0.915
						slm/CA3: *p* = 0.659
						so/CA1: *p* = 0.586
						pcl/CA1: *p* = 0.535
						sr/CA1: *p* = 0.818
						slm/CA1: *p* = 0.971
		VH	*F*(_1,248_) = 1.53,	*F*(_13,248_) = 103.07,	*F*(_13,248_) = 0.15,	o./DG: *p* = 0.918
			*p* = 0.217	***p* < 0.001**	*p* = 0.999	m./DG: *p* = 0.714
						i./DG: *p* = 0.467
						gcl/DG: *p* = 0.382
						pl/DG: *p* = 0.779
						so/CA3: *p* = 0.703
						pcl/CA3: *p* = 0.511
						sl/CA3: *p* = 0.828
						sr/CA3: *p* = 0.674
						slm/CA3: *p* = 0.872
						so/CA1: *p* = 0.653
						pcl/CA1: *p* = 0.95
						sr/CA1: *p* = 0.912
						slm/CA1: *p* = 0.521
mGlu5	1 week	DH	*F*(_1,198_) = 0.87,	*F*(_10,198_) = 5.93,	*F*(_10,198_) = 0.44,	ml/DG: *p* = 0.841
			*p* = 0.351	***p* < 0.001**	*p* = 0.927	gcl/DG: *p* = 0.877
						pl/DG: *p* = 0.904
						so/CA3: *p* = 0.265
						pcl/CA3: *p* = 0.238
						sr/CA3: *p* = 0.507
						slm/CA3: *p* = 0.461
						so/CA1: *p* = 0.86
						pcl/CA1: *p* = 0.943
						sr/CA1: *p* = 0.723
						slm/CA1: *p* = 0.505
		IH	*F*(_1,198_) = 13.08,	*F*(_10,198_) = 7.65,	*F*(_10,198_) = 0.21,	ml/DG: *p* = 0.176
			***p* < 0.001**	***p* < 0.001**	*p* = 0.995	gcl/DG: *p* = 0.285
						pl/DG: *p* = 0.168
						so/CA3: *p* = 0.278
						pcl/CA3: *p* = 0.133
						sr/CA3: *p* = 0.332
						slm/CA3: *p* = 0.375
						so/CA1: *p* = 0.848
						pcl/CA1: *p* = 0.484
						sr/CA1: *p* = 0.506
						slm/CA1: *p* = 0.532
		VH	*F*(_1,198_) = 2.66,	*F*(_10,198_) = 7.34,	*F*(_10,198_) = 0.06,	ml/DG: *p* = 0.745
			*p* = 0.104	***p* < 0.001**	*p* = 0.999	gcl/DG: *p* = 0.497
						pl/DG: *p* = 0.567
						so/CA3: *p* = 0.883
						pcl/CA3: *p* = 0.789
						sr/CA3: *p* = 0.505
						slm/CA3: *p* = 0.468
						so/CA1: *p* = 0.814
						pcl/CA1: *p* = 0.759
						sr/CA1: *p* = 0.696
						slm/CA1: *p* = 0.63
	3 months	DH	*F*(_1,330_) = 20.77,	*F*(_10,330_) = 64.67,	*F*(_10,330_) = 0.71,	ml/DG: *p* = 0.354
			***p* < 0.001**	***p* < 0.001**	*p* = 0.718	gcl/DG: *p* = 0.258
						pl/DG: *p* = 0.619
						so/CA3: *p* = 0.386
						pcl/CA3: *p* = 0.438
						sr/CA3: *p* = 0.426
						slm/CA3: *p* = 0.586
						**so/CA1: *p* = 0.004**
						**pcl/CA1: *p* = 0.03**
						**sr/CA1: *p* = 0.031**
						slm/CA1: *p* = 0.223
		IH	*F*(_1,330_) = 58.18,	*F*(_10,330_) = 21.4,	*F*(_10,330_) = 0.75,	**ml/DG: *p* = 0.043**
			***p* < 0.001**	***p* < 0.001**	*p* = 0.678	**gcl/DG: *p* = 0.037**
						**pl/DG: *p* = 0.005**
						so/CA3: *p* = 0.083
						pcl/CA3: *p* = 0.331
						sr/CA3: *p* = 0.195
						slm/CA3: *p* = 0.408
						**so/CA1: *p* = 0.002**
						**pcl/CA1: *p* = 0.005**
						**sr/CA1: *p* = 0.005**
						**slm/CA1: *p* = 0.024**
		VH	*F*(_1,330_) = 34.16,	*F*(_10,330_) = 48.02,	*F*(_10,330_) = 1.47,	ml/DG: *p* = 0.299
			***p* < 0.001**	***p* < 0.001**	*p* = 0.149	gcl/DG: *p* = 0.377
						pl/DG: *p* = 0.296
						so/CA3: *p* = 0.129
						pcl/CA3: *p* = 0.44
						sr/CA3: *p* = 0.212
						slm/CA3: *p* = 0.675
						**so/CA1: *p* < 0.001**
						**pcl/CA1: *p* = 0.004**
						**sr/CA1: *p* = 0.012**
						slm/CA1: *p* = 0.215


*The table describes the mean ± SEM values. Significant differences are highlighted in bold font. DH, dorsal hippocampus; IH, intermediate hippocampus; VH, ventral hippocampus; ml, molecular layer of the DG; o., outer band of the ml; m., middle band of the ml; i., inner band of the ml; gcl, granule cell layer of the DG; pl, polymorphic layer of the DG; so, Stratum oriens of CA3/CA1; pcl, pyramidal cell layer of CA3/CA1; sl, Stratum lucidum of the CA3; sr, Stratum radiatum of CA3/CA1; and slm, Stratum lacunosum-moleculare of CA3/CA1.*

Three months after MK801-treatment receptor expression was equivalent in hippocampi from MK801- and vehicle-treated animals [multifactorial ANOVA, Treatment factor, DH: *F*(_1,198_) = 0.885, *p* = 0.347; IH: *F*(_1,187_) = 6.962, *p* < 0.01; VH: *F*(_1,194_) = 0.03, *p* = 0.866] (Figure 2B and Table 1).

### MGlu2/3 Levels Are Upregulated 1 Week After MK801 Treatment, With No Overall Changes Evident 3 Months After Treatment

**FIGURE 3:**
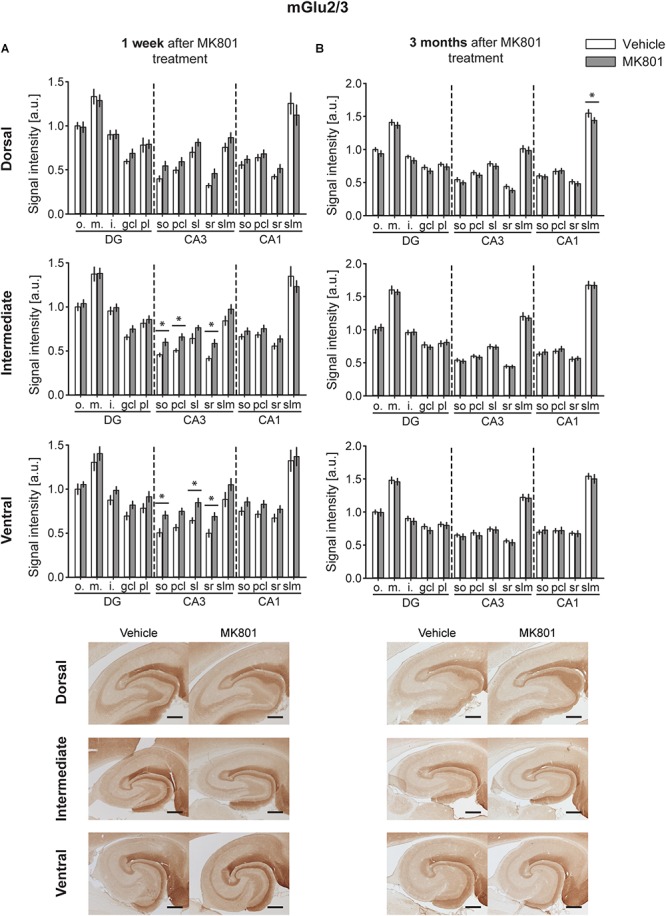
mGlu2/3 receptor levels are transiently up-regulated early after MK801 treatment. Bar charts represent relative changes in protein levels 1 week and 3 months after MK801 treatment across the somato-dendritic layers of the transverse and longitudinal hippocampal axes. **(A)** mGlu2/3 protein expression is (*significantly increased across the ventro-intermediate hippocampal CA3 region 1 week after MK801 treatment. **(B)** No overall changes were detected 3 months after MK801 application. Scale bar: 500 μm. Values expressed in arbitrary units (a.u.). Error bars indicate SEM. ^∗^*p* < 0.05. ml, molecular layer of the DG; gcl, granule cell layer of the DG; pl, polymorphic layer of the DG; so, Stratum oriens of CA3/CA1; pcl, pyramidal cell layer of CA3/CA1; sr, Stratum radiatum of CA3/CA1; and slm, Stratum lacunosum-moleculare of CA3/CA1. Photomicrographs provide examples of mGlu2/3 receptor-stained sections from the dorsal, intermediate and ventral hippocampal subdivisions that originated from the same vehicle- or MK801-treated animal and correspond to 1 week (left) or 3 months (right) after treatment.*)

A significant up-regulation in the levels of mGlu2/3 receptors was found 1 week after MK801 administration in the CA3 region of both the IH and VH [multifactorial ANOVA, Treatment factor, IH: *F*(_1,238_) = 18.317, *p* < 0.001; VH: *F*(_1,238_) = 36.241, *p* < 0.001]. The layers affected included so, pcl, and sr of the IH CA3; and so, sl and sr of the VH CA3 (Figure 3A and Table 1). The DH was unaffected [multifactorial ANOVA, Treatment factor, DH: *F*(_1,238_) = 5.687, *p* < 0.05].

Overall, no changes occurred in mGlu2/3 expression 3 months after treatment [multifactorial ANOVA, Treatment factor, DH: *F*(_1,252_) = 9.8, *p* < 0.01; IH: *F*(_1,252_) = 0.00, *p* = 0.961; VH: *F*(_1,248_) = 1.53, *p* = 0.217]. There was one exception to this, however: we detected a significant decrease in mGlu2/3 expression in the slm of the DH CA1 (Figure 3B and Table 1).

### MGlu5 Expression Is Increased 3 Months After MK801 Application

**FIGURE 4:**
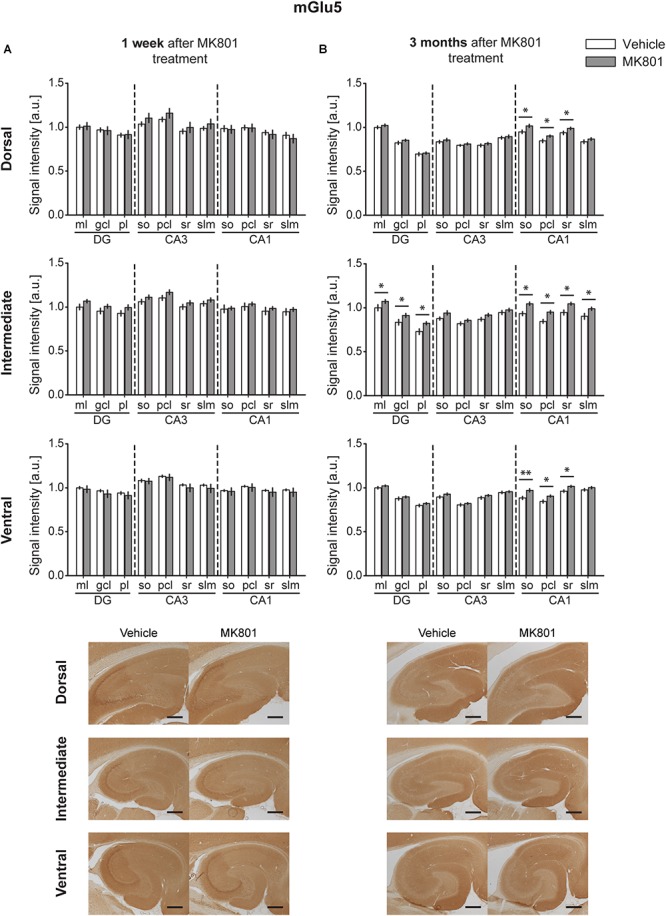
Late up-regulation of mGlu5 receptor levels occurs after MK801 treatment. Bar charts represent relative changes in protein levels 1 week and 3 months after MK801 treatment across the somato-dendritic layers of the transverse and longitudinal hippocampal axes. **(A)** mGlu5 receptor expression remained unaltered (*1 week after MK801 treatment. **(B)** Three months after MK801 injection led to an extensive up-regulation of mGlu5 receptor levels across an entire hippocampus. Scale bar: 500 μm. Values expressed in arbitrary units (a.u.). Error bars indicate SEM. ^∗^*p* < 0.05. ml, molecular layer of the DG; gcl, granule cell layer of the DG; pl, polymorphic layer of the DG; so, Stratum oriens of CA3/CA1; pcl, pyramidal cell layer of CA3/CA1; sr, Stratum radiatum of CA3/CA1; and slm, Stratum lacunosum-moleculare of CA3/CA1. Photomicrographs provide examples of mGlu5 receptor-stained sections from the dorsal, intermediate and ventral hippocampal subdivisions that originated from the same vehicle- or MK801-treated animal and correspond to 1 week (left) or 3 months (right) after treatment.*)

One week after MK801-treatment, mGlu5 receptor expression was unchanged in the hippocampus [multifactorial ANOVA, Treatment factor, DH: *F*(_1,198_) = 0.87, *p* = 0.351; IH: *F*(_1,198_) = 13.08, *p* < 0.001; VH: *F*(_1,198_) = 2.66, *p* = 0.104] (Figure 4A and Table 1). By contrast, 3 months after treatment, a widespread up-regulation of mGlu5 receptor expression was evident across the entire CA1 region (dorsal, intermediate and ventral CA1) and in the intermediate DG compared to controls [multifactorial ANOVA, Treatment factor, DH: *F*(_1,330_) = 20.77, *p* < 0.001; IH: *F*(_1,330_) = 58.18, *p* < 0.001; VH: *F*(_1,330_) = 34.16, *p* < 0.001]. The layers affected included the so, pcl, and sr of the DH CA1, the so, pcl, and sr of the VH CA1, and the so, pcl, sr, and slm of the intermediate CA1. In addition, increased mGlu5 receptor expression occurred in the ml, gcl, and pl of the intermediate DG (Figure 4B and Table 1).

### GABA_A_ Levels Remained Largely Unaltered Following MK801 Treatment

**FIGURE 5:**
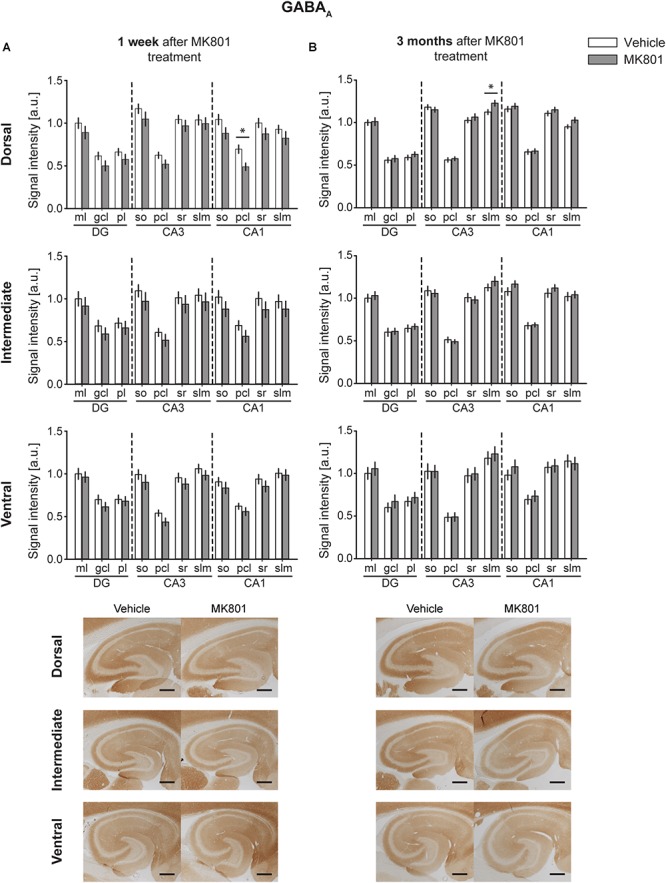
GABA_A_ protein levels display no substantial changes 1 week or 3 months after MK801 treatment. Bar charts illustrate relative changes in receptor protein levels across the somato-dendritic layers of the transverse and longitudinal hippocampal axes. **(A)** No overall changes were detected 1 week after MK801 treatment. **(B)** Similarly, no overall changes were detected 3 months after MK801 treatment. Scale bar: 500 μm. Values expressed in arbitrary units (a.u.). Error bars indicate SEM. ^∗^*p* < 0.05. ml, molecular layer of the DG; gcl, granule cell layer of the DG; pl, polymorphic layer of the DG; so, Stratum oriens of CA3/CA1; pcl, pyramidal cell layer of CA3/CA1; sr, Stratum radiatum of CA3/CA1; and slm, Stratum lacunosum-moleculare of CA3/CA1. Photomicrographs provide examples of GABA_A_ receptor-stained sections from the dorsal, intermediate and ventral hippocampal subdivisions that originated from the same vehicle- or MK801-treated animal, and correspond to 1 week (left) or 3 months (right) after treatment.

One week after MK801 treatment, a potent decrease in GABA_A_ expression occurred in the pcl of the DH CA1 region (Figure 5A). No changes were found in any other subcompartment of the hippocampus [multifactorial ANOVA, Treatment factor, IH: *F*(_1,187_) = 8.805, *p* < 0.01; VH: *F*(_1,183_) = 8, *p* < 0.01] (Figure 5A and Table 2). Three months after treatment a significant increase in GABA_A_ expression was evident in the slm of the DH CA3 region (Figure 5B). Expression levels in all other subcompartments were not different from controls at this time-point [multifactorial ANOVA, Treatment factor, IH: *F*(_1,190_) = 1.52, *p* = 0.219; VH: *F*(_1,198_) = 1.308, *p* = 0.254] (Figure 5B and Table 2).

**Table 2:** An overview of statistically significant differences in the expression of GABAergic receptors.

Receptor	Time-point	Hippocampal axis part	Multifactorial ANOVA			
			TREATMENT factor (T)	REGION factor (R)	T ^∗^ R interaction	Duncan’s *post hoc* test
GABA A	1 week	DH	*F*(_1,176_) = 21.775,	*F*(_10,176_) = 26.225,	*F*(_10,176_) = 0.279,	ml/DG: *p* = 0.233
			***p* < 0.001**	***p* < 0.001**	*p* = 0.985	gcl/DG: *p* = 0.195
						pl/DG: *p* = 0.338
						so/CA3: *p* = 0.134
						pcl/CA3: *p* = 0.247
						sr/CA3: *p* = 0.437
						slm/CA3: *p* = 0.626
						so/CA1: *p* = 0.094
						**pcl/CA1: *p* = 0.027**
						sr/CA1: *p* = 0.187
						slm/CA1: *p* = 0.266
		IH	*F*(_1,187_) = 8.805,	*F*(_10,187_) = 10.303,	*F*(_10,187_) = 0.06,	ml/DG: *p* = 0.521
			***p* < 0.01**	***p* < 0.001**	*p* = 0.999	gcl/DG: *p* = 0.447
						pl/DG: *p* = 0.649
						so/CA3: *p* = 0.349
						pcl/CA3: *p* = 0.459
						sr/CA3: *p* = 0.56
						slm/CA3: *p* = 0.56
						so/CA1: *p* = 0.301
						pcl/CA1: *p* = 0.321
						sr/CA1: *p* = 0.346
						slm/CA1: *p* = 0.494
		VH	*F*(_1,183_) = 8,	*F*(_10,183_) = 22.144,	*F*(_10,183_) = 0.128,	ml/DG: *p* = 0.672
			***p* < 0.01**	***p* < 0.001**	*p* = 0.999	gcl/DG: *p* = 0.332
						pl/DG: *p* = 0.794
						so/CA3: *p* = 0.33
						pcl/CA3: *p* = 0.179
						sr/CA3: *p* = 0.412
						slm/CA3: *p* = 0.402
						so/CA1: *p* = 0.423
						pcl/CA1: *p* = 0.453
						sr/CA1: *p* = 0.338
						slm/CA1: *p* = 0.803
	3 months	DH	*F*(_1,190_) = 6.12,	*F*(_10,190_) = 147.24,	*F*(_10,190_) = 0.7,	ml/DG: *p* = 0.799
			***p* < 0.05**	***p* < 0.001**	*p* = 0.719	gcl/DG: *p* = 0.735
						pl/DG: *p* = 0.385
						so/CA3: *p* = 0.503
						pcl/CA3: *p* = 0.736
						sr/CA3: *p* = 0.416
						**slm/CA3: *p* = 0.033**
						so/CA1: *p* = 0.46
						pcl/CA1: *p* = 0.835
						sr/CA1: *p* = 0.362
						slm/CA1: *p* = 0.109
		IH	*F*(_1,190_) = 1.52,	*F*(_10,190_) = 64.58,	*F*(_10,190_) = 0.44,	ml/DG: *p* = 0.632
			*p* = 0.219	***p* < 0.001**	*p* = 0.927	gcl/DG: *p* = 0.847
						pl/DG: *p* = 0.708
						so/CA3: *p* = 0.662
						pcl/CA3: *p* = 0.714
						sr/CA3: *p* = 0.678
						slm/CA3: *p* = 0.26
						so/CA1: *p* = 0.189
						pcl/CA1: *p* = 0.859
						sr/CA1: *p* = 0.357
						slm/CA1: *p* = 0.714
		VH	*F*(_1,198_) = 1.308,	*F*(_10,198_) = 23.24,	*F*(_10,198_) = 0.139,	ml/DG: *p* = 0.611
			*p* = 0.254	***p* < 0.001**	*p* = 0.999	gcl/DG: *p* = 0.473
						pl/DG: *p* = 0.673
						so/CA3: *p* = 0.983
						pcl/CA3: *p* = 0.975
						sr/CA3: *p* = 0.836
						slm/CA3: *p* = 0.606
						so/CA1: *p* = 0.403
						pcl/CA1: *p* = 0.699
						sr/CA1: *p* = 0.85
						slm/CA1: *p* = 0.748
GABA B	1 week	DH	*F*(_1,198_) = 1.547,	*F*(_10,198_) = 24.281,	*F*(_10,198_) = 0.081,	ml/DG: *p* = 0.873
			*p* = 0.215	***p* < 0.001**	*p* = 0.999	gcl/DG: *p* = 0.554
						pl/DG: *p* = 0.984
						so/CA3: *p* = 0.772
						pcl/CA3: *p* = 0.616
						sr/CA3: *p* = 0.835
						slm/CA3: *p* = 0.368
						so/CA1: *p* = 0.505
						pcl/CA1: *p* = 0.715
						sr/CA1: *p* = 0.738
						slm/CA1: *p* = 0.991
		IH	*F*(_1,198_) = 37.22,	*F*(_10,198_) = 19.34,	*F*(_10,198_) = 0.22,	ml/DG: *p* = 0.065
			***p* < 0.001**	***p* < 0.001**	*p* = 0.993	gcl/DG: *p* = 0.119
						pl/DG: *p* = 0.192
						so/CA3: *p* = 0.0507
						pcl/CA3: *p* = 0.283
						sr/CA3: *p* = 0.136
						**slm/CA3: *p* = 0.019**
						so/CA1: *p* = 0.098
						pcl/CA1: *p* = 0.264
						sr/CA1: *p* = 0.16
						**slm/CA1: *p* = 0.028**
		VH	*F*(_1,187_) = 0.576,	*F*(_10,187_) = 17.222,	*F*(_10,187_) = 0.512,	ml/DG: *p* = 0.81
			*p* = 0.449	***p* < 0.001**	*p* = 0.88	gcl/DG: *p* = 0.573
						pl/DG: *p* = 0.753
						so/CA3: *p* = 0.431
						pcl/CA3: *p* = 0.438
						sr/CA3: *p* = 0.28
						slm/CA3: *p* = 0.259
						so/CA1: *p* = 0.92
						pcl/CA1: *p* = 0.986
						sr/CA1: *p* = 0.913
						slm/CA1: *p* = 0.667
	3 months	DH	*F*(_1,190_) = 44.32,	*F*(_10,190_) = 48.54,	*F*(_10,190_) = 0.55,	ml/DG: *p* = 0.123
			***p* < 0.001**	***p* < 0.001**	*p* = 0.852	pl/DG: *p* = 0.061
						**so/CA3: *p* = 0.03**
						pcl/CA3: *p* = 0.163
						sr/CA3: *p* = 0.378
						slm/CA3: *p* = 0.483
						**so/CA1: *p* = 0.012**
						**pcl/CA1: *p* = 0.009**
						**sr/CA1: *p* = 0.009**
						**slm/CA1: *p* = 0.036**
		IH	*F*(_1,198_) = 1.82,	*F*(_10,198_) = 34.01,	*F*(_10,198_) = 0.18,	ml/DG: *p* = 0.914
			*p* = 0.178	***p* < 0.001**	*p* = 0.997	gcl/DG: *p* = 0.824
						pl/DG: *p* = 0.907
						so/CA3: *p* = 0.214
						pcl/CA3: *p* = 0.44
						sr/CA3: *p* = 0.82
						slm/CA3: *p* = 0.851
						so/CA1: *p* = 0.579
						pcl/CA1: *p* = 0.893
						sr/CA1: *p* = 0.657
						slm/CA1: *p* = 0.955
		VH	*F*(_1,187_) = 0.19,	*F*(_10,187_) = 24.44,	*F*(_10,187_) = 0.2,	ml/DG: *p* = 0.811
			*p* = 0.662	***p* < 0.001**	*p* = 0.995	gcl/DG: *p* = 0.737
						pl/DG: *p* = 0.727
						so/CA3: *p* = 0.629
						pcl/CA3: *p* = 0.988
						sr/CA3: *p* = 0.866
						slm/CA3: *p* = 0.955
						so/CA1: *p* = 0.947
						pcl/CA1: *p* = 0.634
						sr/CA1: *p* = 0.941
						slm/CA1: *p* = 0.305


The table describes the mean ± SEM values. Significant differences are highlighted in bold font. DH, dorsal hippocampus; IH, intermediate hippocampus; VH, ventral hippocampus; ml, molecular layer of the DG; gcl, granule cell layer of the DG; pl, polymorphic layer of the DG; so, Stratum oriens of CA3/CA1; pcl, pyramidal cell layer of CA3/CA1; sr, Stratum radiatum of CA3/CA1; and slm, Stratum lacunosum-moleculare of CA3/CA1.

### GABA_B_ Expression Is Downregulated After MK801 Application

**FIGURE 6:**
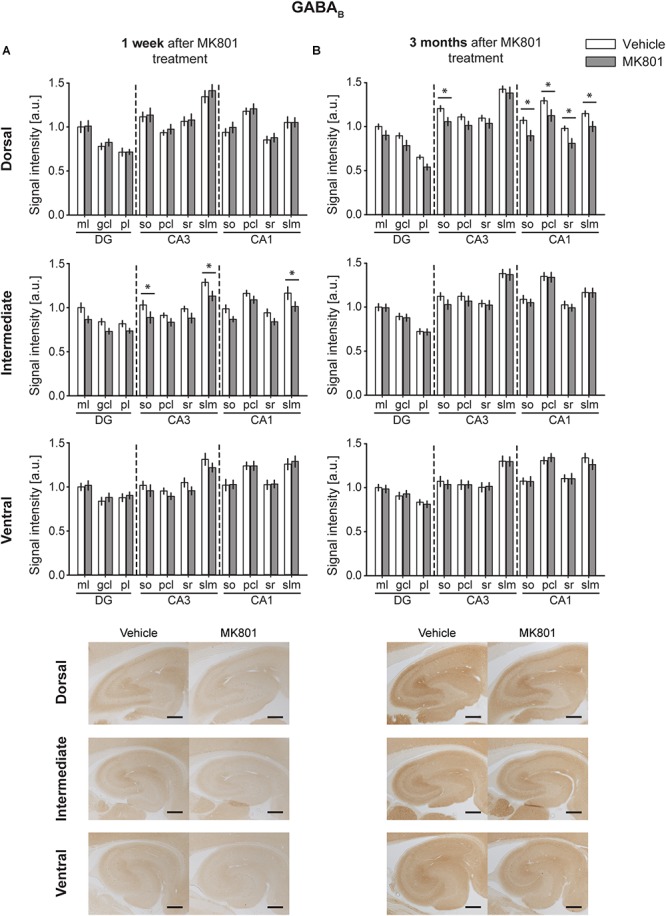
MK801 treatment leads to a down-regulation of GABA_B_ receptor protein expression. Bar charts illustrate relative changes in receptor protein levels across the somato-dendritic layers of the transverse and longitudinal hippocampal axes. **(A)** A reduction of GABA_B_ receptor expression was evident across the CA (*regions of the intermediate hippocampus 1 week after MK801 treatment. **(B)** Similarly, a reduction of GABA_B_ receptor levels was detected across the dorsal CA regions 3 months after MK801 treatment. Scale bar: 500 μm. Values expressed in arbitrary units (a.u.). Error bars indicate SEM. ^∗^*p* < 0.05. ml, molecular layer of the DG; gcl, granule cell layer of the DG; pl, polymorphic layer of the DG; so, Stratum oriens of CA3/CA1; pcl, pyramidal cell layer of CA3/CA1; sr, Stratum radiatum of CA3/CA1; and slm, Stratum lacunosum-moleculare of CA3/CA1. Photomicrographs provide examples of GABA_B_ receptor-stained sections from the dorsal, intermediate and ventral hippocampal subdivisions that originated from the same vehicle- or MK801-treated animal, and correspond to 1 week (left) or 3 months (right) after treatment.*)

One week following MK801 treatment, GABA_B_ receptor changes occurred in the IH compared to controls (Figure 6A) [multifactorial ANOVA, Treatment factor, IH: *F*(_1,198_) = 37.22, *p* < 0.001]. Here, a significant down-regulation was seen in the so and slm of the IH CA3 and in the slm of the IH CA1 (Figure 6A and Table 2). Receptor expression in the DH and VH was equivalent to controls [multifactorial ANOVA, Treatment factor, DH: *F*(_1,198_) = 1.547, *p* = 0.215; VH: *F*(_1,187_) = 0.576, *p* = 0.449] (Table 2).

Three months after MK801 treatment, no receptor changes were evident in the IH or VH [multifactorial ANOVA, Treatment factor, IH: *F*(_1,198_) = 1.82, *p* = 0.178; VH: *F*(_1,187_) = 0.19, *p* = 0.662] (Table 2), but a significant down-regulation in GABA_B_ receptor levels was found across the entire DH cornus ammonis (Figure 6B): All layers (so, pcl, sr, and slm) of the CA1 and CA3 regions were significantly affected (Table 2).

### MK801 Treatment Reduces the Amount of GAD67+ Cells in the Hippocampus

**FIGURE 7:**
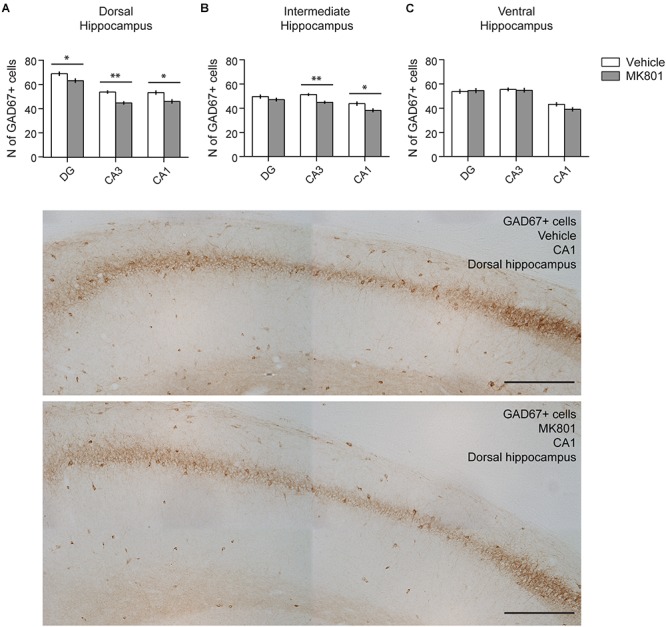
The amount of GAD67+ cells is reduced in the hippocampus after MK801 treatment. Bar charts summarize the number of interneurons 3 months after MK801 treatment across the transverse and longitudinal hippocampal axes: **(A)** dorsal, **(B)** intermediate, **(C)** ventral hippocampus. Whereas significant decreases were detected in subregions of the dorsal and intermediate hippocampus, no changes were observed in the ventral subdivision. Error bars indicate SEM. ^∗^*p* < 0.05, ^∗∗^*p* < 0.01. Examples of GAD67-stained sections from the dorsal, intermediate and ventral hippocampal subdivisions are shown below. Specifically, the CA1 region from vehicle and MK801-treated animals is shown. Scale bar: 300 μm.

Our finding that GABA_B_ receptor expression was potently downregulated 3 months after MK801-treatment might be explained by a loss of interneurons. To check this possibility, we assessed the number of GAD-expressing interneurons in the hippocampus 3 months after MK801 application (Figure 7). Here, we found a significant reduction in the amount of GAD67-positive cells in the DG (*t*-test, Vehicle: 69.21 ± 1.86 vs. MK801: 63.45 ± 1.86, *p* = 0.03, *t* = 2.185 with 90 degrees of freedom), CA3 (*t*-test, Vehicle: 54.06 ± 1.43 vs. MK801: 45.08 ± 1.4, *p* < 0.001, *t* = 4.471 with 88 degrees of freedom), and CA1 (*t*-test, Vehicle median: 52 vs. MK801 median: 45, Mann–Whitney *U* = 668, *n*_V ehicle_ = 44, *n*_MK801_ = 47, *p* = 0.004) regions of the DH and in the CA3 (*t*-test, Vehicle: 51.27 ± 1.14 vs. MK801: 44.86 ± 1.27, *p* < 0.001, *t* = 3.736 with 88 degrees of freedom) and CA1 (*t*-test, Vehicle median: 41.5 vs. MK801 median: 36, Mann–Whitney *U* = 719, *n*_V ehicle_ = 44, *n*_MK801_ = 46, *p* = 0.01) regions of the IH. No changes could be observed in the VH (*t*-test, DG, Vehicle: 53.78 ± 1.96 vs. MK801: 54.53 ± 2.08, *p* = 0.79, *t* = -0.263 with 83 degrees of freedom; CA3, Vehicle: 55.53 ± 1.55 vs. MK801: 54.84 ± 1.97, *p* = 0.78, *t* = 0.276 with 85 degrees of freedom), although a trend toward a decrease was detected in the CA1 region (*t*-test Vehicle: 43.19 ± 1.8 vs. MK801: 39.09 ± 1.75, *p* = 0.1, *t* = 1.628 with 84 degrees of freedom).

## Discussion

In this study, we demonstrate that acute induction of hypoglutamatergic signaling in rats persistently alters the expression of several metabotropic glutamatergic and GABAergic receptors across the hippocampus. Importantly, the profile of rapid (1 week) changes differs from subsequent (3 month) changes. These alterations in receptor expression are also specific to the region (longitudinal axis), subregion (transverse axis) and layer of the hippocampus. In addition, 3 months after treatment, a significant reduction in GAD67-positive interneurons is evident in both the dorsal and the intermediate hippocampus.

In line with evidence for disturbed glutamatergic neurotransmission in schizophrenia and psychosis (Lesage and Steckler, 2010; Lin et al., 2012; Matosin and Newell, 2013), we observed an early impairment in the expression of mGlu1 receptors across the dorso-intermediate hippocampal axis, as well as an upregulation of expression in the intermediate hippocampus. An early up-regulation in mGlu2/3 levels across the ventro-intermediate CA3 region also occurred, whereas GABA_B_ receptor expression was reduced in the intermediate hippocampus. Three months after MK801-treatment, an increase in mGlu5 expression was evident across the entire CA1 region and intermediate DG. At the same time-point a down-regulation in the levels of GABA_B_ receptors occurred across the dorsal hippocampus. These changes can be expected to affect information transfer along the longitudinal axis of the hippocampus (Tóth and Freund, 1992; Andersen et al., 2000), and contribute to disturbed hippocampal-prefrontal cortex communication in psychosis (Heckers et al., 1998; Grüter et al., 2015; Hartung et al., 2016).

The changes in receptor expression that occurred 1 week after MK801-treatment may reflect a rapid adaptive response of the brain to the first episode of psychosis. This in turn, may contribute to the pathological steps that increase brain vulnerability toward disease progression and establishment. These changes are transient, however. Three months after treatment, expression of GABA_A_, mGlu1, and mGlu2/3 receptors had largely normalized, with the exception of a focal decrease of mGlu2/3 receptor expression in the Stratum lacunosum-moleculare of the dorsal CA1 region and a focal elevation of GABA_A_ expression in the Stratum lacunosum-moleculare of the dorsal CA3 region. At this time-point, an extensive elevation of mGlu5 receptor expression had developed across the CA1 region of the entire dorsoventral axis. Strikingly, this result matches a recent post-mortem finding of increased expression of mGlu5 receptors in the hippocampal CA1 region of subjects with schizophrenia (Matosin et al., 2015), possibly suggesting an established phenotype of receptor changes in our animal model 3 months after MK801 administration.

The rodent model of a psychosis-like event that we used here involves an acute treatment of adults rats with the NMDAR antagonist, MK801 (Wozniak et al., 1996; Manahan-Vaughan et al., 2008a,b; Wiescholleck and Manahan-Vaughan, 2013a,b). It triggers widespread changes in NMDAR expression across the hippocampus (Dubovyk and Manahan-Vaughan, 2018b). NMDAR hypofunction in GABAergic interneurons appears to be an important component of models defining impairments in both, humans with psychosis and in relevant animal models (Belforte et al., 2010; Nakazawa et al., 2012; Snyder and Gao, 2013; Cohen et al., 2015; Jézéquel et al., 2018). It offers an explanation for a wide-range of schizophrenia’s positive, negative and cognitive symptoms. A primary alteration in glutamate-GABA levels and consequent destabilization of the levels of other neurotransmitters throughout the brain are believed to underlie these effects (Snyder and Gao, 2013; Perez and Lodge, 2014). Both glutamatergic and GABAergic neurotransmission are linked to a multitude of intrinsic sensory and cognitive processes such as perception, learning and memory (Kaila, 1994; Paulsen and Moser, 1998; Bettler et al., 2004; Malenka and Bear, 2004; Kullmann et al., 2005; Mukherjee and Manahan-Vaughan, 2013; Kesner and Rolls, 2015). Interestingly, both metabotropic glutamate and GABAergic receptors modulate NMDAR function, as well as glutamate and GABA release (Tu et al., 1999; Benquet et al., 2002; Heidinger et al., 2002; Marino and Conn, 2002; Bettler et al., 2004; Kullmann et al., 2005).

The changes in GABA_B_ receptor expression that we detected, is in line with a reported decrease in GABA_B_ receptor expression in the hippocampus of patients suffering from schizophrenia (Mizukami et al., 2000) and is especially interesting given the reported role of GABA_B_ receptors in maintaining neuronal homeostasis in hippocampal networks (Vertkin et al., 2015). GABA_B_ receptors regulate neuronal population firing rate through sensing of ambient GABA levels. This is then transduced into homeostatic changes in synaptic strength (Gassmann and Bettler, 2012; Degro et al., 2015). In addition to the loss of GABA_B_ receptors, 3 months after MK801 treatment, a significant loss of hippocampal interneurons was also detected in our study. A loss of interneurons combined with a decrease of GABA_B_ receptors can be expected to result in a loss of experience-dependent GABA release, of GABA sensitivity and inhibitory tonus in the hippocampus. In line with this, a decrease in GABA_B_ receptors is associated with aberrantly increased neuronal activity in rodents (Schuler et al., 2001; Gassmann et al., 2004). Furthermore, elevations in hippocampal excitability, coupled to aberrant somatic immediate early gene expression and deficits in hippocampus-dependent learning occur several weeks after MK801 treatment of rats (Grüter et al., 2015).

Both mGlu1 (Aiba et al., 1994; Naie and Manahan-Vaughan, 2005; Gil-Sanz et al., 2008; Neyman and Manahan-Vaughan, 2008) and mGlu5 receptors are required for the persistence of hippocampal synaptic plasticity and hippocampus-dependent spatial memory (Naie and Manahan-Vaughan, 2004; Manahan-Vaughan and Braunewell, 2005; Popkirov and Manahan-Vaughan, 2011). In the hippocampus, mGlu1 receptors are predominantly expressed on interneurons (Baude et al., 1993; Ferraguti et al., 1998, 2004). Their activation evokes slow oscillatory inward currents and elevates the frequency of spontaneous inhibitory postsynaptic currents and their amplitude in principal cells (van Hooft et al., 2000; Mannaioni et al., 2001; Lapointe et al., 2004). Hippocampal interneurons dampen neuronal excitability via mGlu1 receptor signaling that increases activation of interneurons and GABA release, (van Hooft et al., 2000; Lapointe et al., 2004). Furthermore, mGlu5 receptors support information transfer through neuronal oscillations in the hippocampus (Bikbaev et al., 2008; Bikbaev and Manahan-Vaughan, 2017). Thus, the changes in expression of these receptors may underlie changes in synaptic plasticity and neuronal oscillations that have been described in the rodent model studied here (Kalweit et al., 2017), as well neuronal oscillations in patients who have experienced a first-episode (Uhlhaas and Singer, 2010).

‘Synaptic gain control’ determines how neurons respond to their inputs as well as the intrinsic excitation-inhibition balance in neural networks (Díez et al., 2017). We propose that changes in the relative balance and distribution of GABA and glutamate receptors underlie the loss of synaptic gain control that has been previously described in this animal model of psychosis (Grüter et al., 2015; Kalweit et al., 2017) and may possibly comprise a fundamental mechanism underlying loss of synaptic gain control as proposed to occur in patients suffering from psychosis (Adams et al., 2013; Ranlund et al., 2016; Díez et al., 2017).

Of the hippocampal subdivisions affected by MK801 treatment, the dorsal hippocampus was the most vulnerable and the ventral hippocampus was the most resistant. Functionally, the convergence of long-term receptor changes in the dorsal hippocampal CA1 region suggests that impairments in memory retrieval and prediction error function will develop (Schultz and Dickinson, 2000). This means that mismatches will occur between external sensory information and internal representations (Hasselmo, 2005; Ji and Maren, 2008). In our animal model, this is reflected by impairments of object recognition memory, object-place memory, sensory gating and spatial reference memory (Grüter et al., 2015; Manahan-Vaughan et al., 2008a). In psychotic patients, this might be reflected by attention and motivation being applied to unimportant events, the formation of erroneous thoughts, disrupted cognition, and possibly delusions (Corlett et al., 2009; Schobel et al., 2013).

In summary, our findings suggest that in an animal model of psychosis that is accompanied by both short-term changes in NMDAR expression and long-term NMDAR hypofunction (Dubovyk and Manahan-Vaughan, 2018a), rapid changes in the expression of metabotropic glutamate receptors and GABA receptors are superseded by an extensive long-term upregulation of mGlu5 receptors in the CA1 regions of the entire dorsoventral hippocampal axis, along with a decrease of GABA_B_ receptor expression across all layers of the dorsal CA1 region and a loss of interneurons. The CA1 region is arguably the output structure of the hippocampus that engages in mismatch detection and has been proposed to be especially vulnerable to NMDAR hypofunction (Lisman and Otmakhova, 2001). Our findings indicate that this structure is particularly susceptible to changes elicited by the instigation of a first episode psychosis-like state in rodents that in turn triggers receptor expression changes that are similar to those reported in schizophrenic patients. We propose that these chronic changes in glutamatergic and GABAergic receptor expression comprise a primary mechanism underlying loss of synaptic gain control that is believed to underlie many features of psychosis including functional disconnectivity, abnormal neuronal oscillations and loss of sensory attenuation (Adams et al., 2013; Díez et al., 2017).

## Ethics Statement

This study was carried out in accordance with the European Communities Council Directive of September 22nd, 2010 (2010/63/EU) for care of laboratory animals. Prior permission was obtained from the local authorities (Landesamt für Arbeitsschutz, Umwelt- und Naturschutz, Nordrhein-Westfalen).

## Author Contributions

DM-V created the concept and strategy of the study. VD carried out the experiments. DM-V and VD conducted data analysis and interpretation, as well as the writing of the manuscript.

## Conflict of Interest Statement

The authors declare that the research was conducted in the absence of any commercial or financial relationships that could be construed as a potential conflict of interest.
